# Molecular phylogeny of *Hiptage* (Malpighiaceae) reveals a new species from Southwest China

**DOI:** 10.3897/phytokeys.135.37011

**Published:** 2019-12-05

**Authors:** Ke Tan, Hai-Lei Zheng, Shu-Peng Dong, Ming-Xun Ren

**Affiliations:** 1 Key Laboratory of Genetics and Germplasm Innovation of Tropical Special Forest Trees and Ornamental Plants, College of Ecology and Environment, Hainan University, Haikou 570228, China Hainan University Haikou China; 2 Wild Dali Nature Education and Research Center, Dali 671000, Yunnan, China Wild Dali Nature Education and Research Center Dali China; 3 Center for Terrestrial Biodiversity of the South China Sea, Hainan University, Haikou 570228, China Hainan University Hainan China

**Keywords:** Asia, *Hiptage incurvatum*, Malpighiales, taxonomy, tetrapteroid clade

## Abstract

*Hiptage* is an Asia-endemic genus of Malpighiaceae currently placed in the tetrapteroid clade, representing one of the seven inter-continent dispersions from New to Old World. A molecular phylogeny based on sequences of the internal transcribed spacer (ITS) region was recovered for the first time for the genus. Our results showed that the most recent common ancestor of *Hiptage* probably originated in the South Indo-China Peninsula and diversified in this region. Based on phylogenetic evidence and relevant morphological traits, we propose a new species; *Hiptage incurvatum* is characterised by mericarps with arcuate anterior lateral wings, two large glands on the dorsal sepals, and small glands on the remaining sepals. The new species is from Mt. Cangshan, Dali City (25°35'N, 100°02'E) in North Yunnan, Southwest China and is notable for its occurrence at high altitude, 1400 m (the highest distribution currently known for the genus). The implications of this unusual species for the dispersal and evolution of the genus are discussed.

## Introduction

*Hiptage* Gaertn. is a genus of Malpighiaceae currently comprising ca. 40 species (Anderson et al. 2006; Chen and Funston 2008; Ren 2015; Yang et al. 2018). Its species typically grow as woody lianas in the margins of evergreen and seasonal rainforests, in river valleys, or on limestone hills of South and Southeast Asia (Sirirugsa 1991; Srivastava 1992; Chen and Funston 2008). The genus is characterised by its many-flowered thyrse bearing mirror-image flowers with nectar secreting sepal glands, and a single mericarp per flower bearing three lateral wings (Anderson et al. 2006; Ren et al. 2013; Zhang et al. 2016). Mirror-image flowers are a sexual polymorphism in which the style is deflected away from the floral axis, resulting in mirror images between the left-styled flower and right-styled flowers, facilitating cross-pollination (Jesson and Barrett 2002; Ren et al. 2013). Currently, the genus is placed in the tetrapteroid clade, one of the ten major lineages recovered for Malpighiaceae by Davis and Anderson (2010). *Hiptage* was represented in that phylogeny by six species and recovered as sister to *Flabellariopsis* R.Wilczek, an African endemic, and together both genera form a poorly supported clade, sister to the Neotropical genus *Carolus* W.R.Anderson (Davis and Anderson 2010). However, the monophyly of *Hiptage* has never been properly tested by the inclusion of its type species (Jacobs 1955; Davis and Anderson 2010; Zhang et al. 2016).

During recent field studies addressing the pollination ecology of *Hiptage* in North Yunnan, two populations of an unusual morphotype of *Hiptage benghalensis* were discovered near Pingpo Town, in Mount Cangshan near Dali City. After molecular and morphological analyses, based on the nuclear internal transcribed spacer (ITS) region and on the comparison of living and herbarium specimens (including type specimens of all currently accepted names in the genus), we concluded that these abovementioned populations represent an undescribed species of *Hiptage*. We present a molecular phylogeny sampling 17 of 39 species of *Hiptage*, including a discussion on the systematics and biogeography of the genus, besides the formal description of the new species and an updated key for the genus in China.

## Materials and methods

### Molecular analysis

We sampled most species of *Hiptage* occurring in the Philippines, Thailand, Vietnam, Singapore and Southwest China, to explore the phylogenetic relationships of the suspected new species (Table 1). The sequences of nuclear ribosomal Internal Transcribed Spacer (ITS) region of 17 species (with some species with multi accessions) of *Hiptage* were generated and analysed. The ITS sequences of two American-endemic species of *Mascagnia* (i.e., *M. australis* and *M. divaricata*) were obtained from GenBank and used as outgroups. Total genomic DNA was extracted from dried leaf material following a modified CTAB method (Doyle and Doyle 1987). All polymerase chain reactions (PCR) were carried out in 25 μl volumes consisting of 1 μl sample DNA, 12.5 μl 2 × Taq PCR master Mix (Aidlab Biotechnologies Co. Ltd), 1 μl each primer (10 μmol/ml), and a final volume adjusted to 25 μl with double distilled water. The ITS region was amplified with the primers ITS17SE and ITS26SE (Sun et al. 1994). We used an amplification profile with an initial denaturation of 5 min at 94 °C, followed by 35 cycles of 40 seconds at 94 °C, 20 seconds at 69 °C, 1 min at 72 °C, and a final 10 min extension at 72 °C. The PCR products were sequenced from both directions using an ABI3730XL sequencer.

**Table 1.** Taxa and GenBank accession numbers for the nrITS sequences used in this study; an asterisk (*) indicates the new species record.

Species	Locality	GenBank accession numbers	Voucher number
*Hiptage benghalensis* (L.) Kurz	Phatthaya, Thailand	MH718408	K. Tan, S. P. Dong, & M. X. Ren 3344 (HUTB)
	Chiang Mai, Thailand	MH718410	K. Tan, S. P. Dong, & M. X. Ren 3336 (HUTB)
	Singapore	MH718399	T. W. Yam 3334 (HUTB)
	Lekang County, Guizhou, China	MH718415	K. Tan, S. P. Dong, & M. X. Ren 82 (HUTB)
	Yangjie, Yunnan, China	MH718400	M. X. Ren & L. Tang 128 (HUTB)
	Daxin County, Guangxi, China	MH718414	K. Tan & S. P. Dong 95 (HUTB)
	Menglian County, Yunnan, China	MH718422	S. P. Dong 131 (HUTB)
*H. bullata* Craib	Lampang, Thailand	MH718412	K. Tan, S. P. Dong, & M. X. Ren 3320 (HUTB)
*H. candicans* Hook. f.	Chiang Mai, Thailand	MH718409	K. Tan, S. P. Dong, & M. X. Ren 3328 (HUTB)
	Chom Thong, Thailand	MH718411	K. Tan, S. P. Dong, & M. X. Ren 3330 (HUTB)
*H. detergens* Craib	Kui Buri, Thailand	MH718404	K. Tan, S. P. Dong, & M. X. Ren 3328 (HUTB)
	Sam Roi Yot, Thailand	MH718405	K. Tan, S. P. Dong, & M. X. Ren 3326 (HUTB)
*H. ferruginea* Y.H.Tan & Bin Yang	Xishuangbanna, Yunnan, China	MH718402	S. P. Dong 116 (HUTB)
	Xishuangbanna, Yunnan, China	MH718403	S. P. Dong 117 (HUTB)
*H. incurvatum* 1*	Pingpo Town, Yunnan, China	MK967956	K. Tan, H. L. Zheng, & M. X. Ren 201903309 (HUTB)
*H. incurvatum* 2*	Pingpo Town, Yunnan, China	MK967957	K. Tan, H. L. Zheng, & M. X. Ren 201903310 (HUTB)
*H. incurvatum* 3*	Pingpo Town, Yunnan, China	MK967958	K. Tan, H. L. Zheng, & M. X. Ren 201903305 (HUTB)
*H. incurvatum* 4*	Pingpo Town, Yunnan, China	MK967959	K. Tan, H. L. Zheng, & M. X. Ren 201903306 (HUTB)
*H. lucida* Pierre	Phatthaya, Thailand	MH718406	K. Tan, S. P. Dong, & M. X. Ren 38 (HUTB)
	Xishuangbanna, Yunnan, China	MH718418	Z. N. Qian & S. P. Dong120 (HUTB)
*H. luzonica* Merr.	Luzon Island, Philippines	MH718425	K. Tan, W. Q. Xiang & M. X. Ren 20191181436 (HUTB)
	Cebu Island, Philippines	MH718431	K. Tan & W. Q. Xiang 3301(HUTB)
	Palawan Island, Philippines	MH718432	K. Tan, W. Q. Xiang & M. X. Ren 3305 (HUTB)
*H. marginata* Arènes	Hue, Vietnam	MH718413	K. Tan & Q. Yang 3363 (HUTB)
*H. minor* Dunn	Lushui City, Yunnan, China	MH718401	K. Tan, S. P. Dong, & M. X. Ren 88 (HUTB)
	Lekang County, Guizhou, China	MH718398	K. Tan, S. P. Dong, & M. X. Ren 79 (HUTB)
	Wenshan City, Yunnan, China	MH718423	K. Tan, S. P. Dong, & M. X. Ren 94 (HUTB)
*H. monopteryx* Sirirugsa	Phatthaya, Thailand	MH718407	K. Tan, S. P. Dong, & M. X. Ren 3337 (HUTB)
*H. multiflora* F.N.Wei	Nonggang Natural Reserve, Guangxi, China	MH718424	K. Tan & S. P. Dong 52 (HUTB)
*H. pauciflora* Y.H.Tan & Bin Yang	Menglian County, Yunnan, China	MH718420	S. P. Dong 73 (HUTB)
*H. stellulifera* Arènes	Nha Trang, Vietnam	MH718429	K. Tan & S. J. Ling 3376 (HUTB)
*H. subglabra* Arènes	Nui Chua National Park, Phan Rang, Vietnam	MH718427	K. Tan & S. J. Ling 3364 (HUTB)
*H. tianyangensis* F.N.Wei	Liulian Town, Tianyang, Guangxi, China	MK967960	K. Tan & S. P. Dong 50 (HUTB)
*H. umbellulifera* Arènes	Nui Chua National Park, Phan Rang, Vietnam	MH718428	K. Tan & S. J. Ling 3385 (HUTB)
	Cana, Phan Rang, Vietnam	MH718426	K. Tan & S. J. Ling 3386 (HUTB)
	Phan Rang, Vietnam	MH718430	K. Tan & S. J. Ling 3399 (HUTB)
*Mascagnia australis* C.E. Anderson	South America	KR092931	A. Francener 1177 (SP)
*M. divaricata* (Kunth) Nied.	South America	KR092932	R. F. Almeida 547 (HUEFS)

The original chromatograms from both directions of the ITS sequences were evaluated with PhyDE (Müller et al. 2010) for base confirmation and contiguous sequences editing. All sequences were aligned manually in MEGA v.7 (Kumar et al. 2016). Ambiguous positions were excluded from the alignments. The Akaike Information Criterion (AIC), which allows non-nested models to be evaluated, was used as a selection criterion (Kumar et al. 2016), and the GTR + I + G model was used in both ML and BI analyses. Maximum Likelihood (ML) analysis was performed with optimal substitution models suggested by MEGA v.7 to carry out 1000 bootstrap (BS) replicates analyses. Bayesian inference (BI) was performed with MrBayes v.3.1 (Ronquist and Huelsenbeck 2003) with a Markov chain Monte Carlo (MCMC) simulations were run for 10 000 000 generations and sampled every 1000 generations. The first 2500 trees (25% of total trees) were discarded as burn-in. The remaining trees were summarised in a 50% majority-rule consensus tree, and the posterior probabilities (PP). The obtained tree was edited using Figtree v. 1.4.3 (Morariu et al. 2008). Sequences were deposited in GenBank and the alignment and phylogenetic trees in TreeBASE (ID: S24963 and S24968).

### Taxonomy

The proposed new species was compared with the type specimens of all accepted names in the genus, including collections of *Hiptage* deposited in the herbaria KUN, PE, IBSC, and IBK (acronyms according to Thiers 2019). We also downloaded all *Hiptage* specimens from JSTOR Global Plants (http://plants.jstor.org), and Chinese Virtual Herbarium (http://www.cvh.ac.cn) to compare detailed morphological traits between the proposed new species with the currently accepted species of *Hiptage*. The morphological terminology follows Niedenzu (1924), Jacobs (1955), Hô (1992), Anderson et al. (2006), Chen and Funston (2008), Pelser et al. (2011), and Ren (2015).

## Results

For the 17 *Hiptage* species, we obtained 36 sequences of ITS in total. Source information and the GenBank accession numbers of the new sequences are listed in Table 1. The dataset had an aligned length of 691 base pairs (bp), containing 128 parsimony-informative characters.

**Figure 1.**
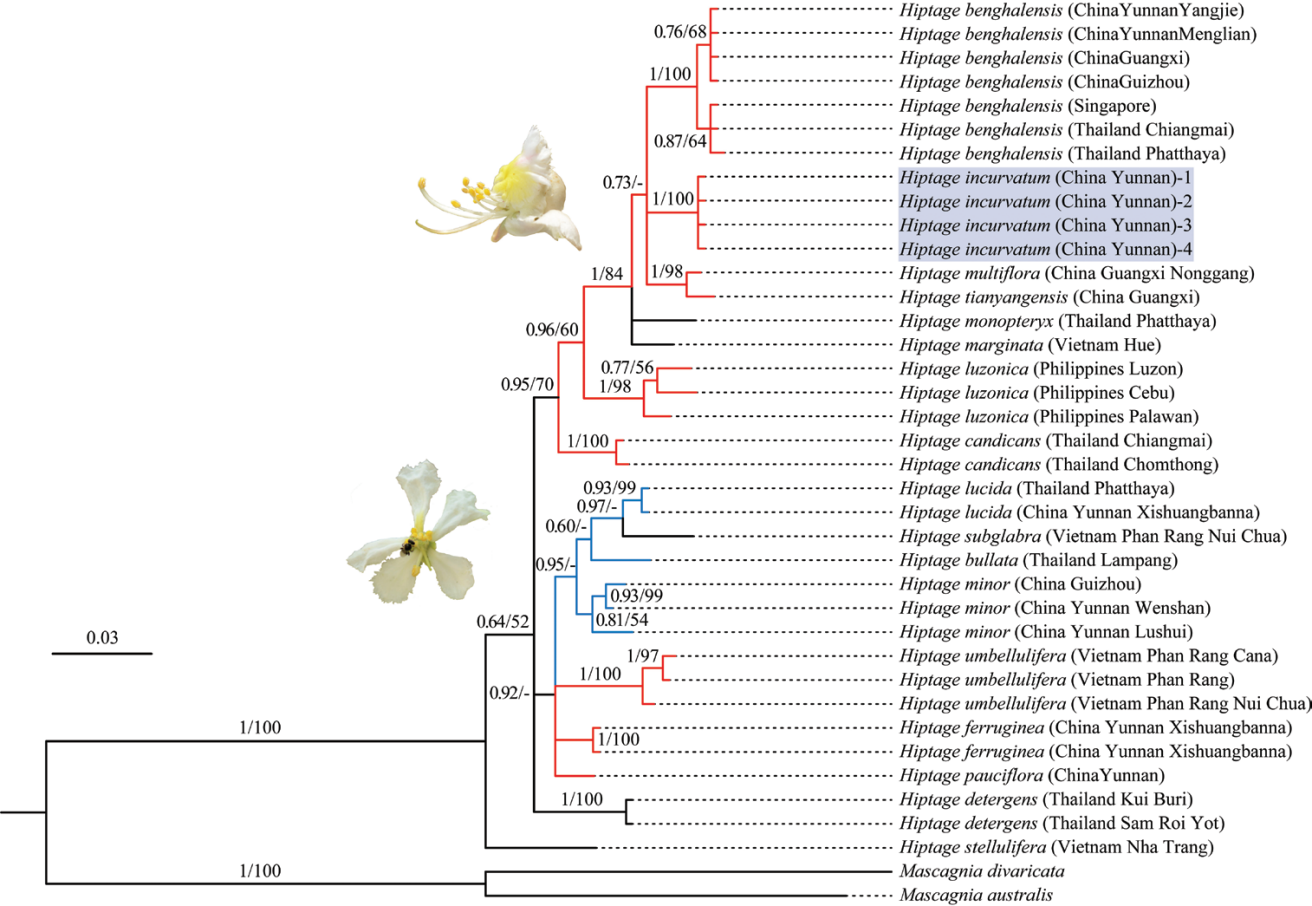
Molecular phylogeny for 17 species of *Hiptage* and two Neotropical outgroups based on ITS sequences. Bayesian posterior probability (PP) and MP bootstrap values (BS) are showed above branches as PP/BS (only shown if BS > 50%). *H. incurvatum* was shown in grey. The red, blue, black clades indicate reflexed petals, erect petals, and unknown, respectively. Inserted photos indicate petal-reflexed flowers (red branches) and petal-plat flowers (blue branches). Black branches represent the unclear mode.

In the ITS tree (Fig. 1) *Hiptage* was recovered as monophyletic, strongly supported (PP/BS=1/100) by both analyses, with *H. stellulifera* from Vietnam as the first diverging lineage in the genus. The remaining species of *Hiptage* sampled formed two separate clades, although with weak support (PP/BS=0.64/52). Most species of *Hiptage* show reflexed petals (red line), with all species bearing erect petals (i.e., *H. lucida*, *H. bullata*, and *H. minor*) being recovered on a single clade, suggesting a single origin of erect petals in the genus (Fig. 1). The four specimens of the proposed new species, *H. incurvatum*, coalesced in a clade with strong support (PP/BS=1/100%) (Fig. 1). This clade was recovered in a polytomy, the *H. multiflora* + *H. tianyangensis* clade, from Guangxi Province, and the widespread *H. benghalensis* clade, from Southwest China and Indo-China Peninsula (Fig. 1). Relationships among these four species are mostly poorly supported (PP/BS=0.73/-) with the exception of the strongly supported *H. multiflora* + *H. tianyangensis* clade (PP/BS=1/98).

### Taxonomy

#### Hiptage incurvatum

Taxon classificationPlantaeMalpighialesMalpighiaceae

K.Tan & M.X.Ren sp. nov.

775021D4-216A-5C75-886B-836D996F8A5C

urn:lsid:ipni.org:names:77203327-1

Figs 2 , 3

##### Type.

China. Yunnan Province: Pingpo Town, Mt. Cangshan, Dali City, 25°35'N, 100°02'E, 1400 m altitude. 31 Mar 2019, *K. Tan and M.X. Ren 2019033110* (holotype: HUTB!, isotypes: HUTB!, KUN!)

##### Diagnosis.

Similar to *H. tianyangensis* in ovate leaf shape, suborbicular petals; but differing from this species by sepal glands twice big as *H. tianyangensis* (vs. sepal gland, ~ 3 × 1 mm), the elevation ca. 400 m (vs. 1379–1724 m), the short inflorescence 1–4 cm (vs. 4–10 cm).

##### Description.

Woody lianas; stems 20–30 (–200) mm diam. Branches round, lenticels white or greenish, tomentose to glabrous, with white to grey hairs. Leaves opposite; stipules absent; petioles ca 0.5 cm long, round, tomentose, with white hairs, eglandular; leaf blades 6–12 × 2.5–4.5 cm, elliptic, base cuneate, margin plane, apex attenuate, both surfaces sericeous, 10–16-glandular dots abaxially near margin, lateral veins 5–8 pairs, prominent on both surfaces. *Thyrses*, solitary, axillary or terminal; main axis 4–10 cm long, tomentose, with white hairs; peduncles 1.5–2.5 cm long, tomentose; bracteoles inserted below the apex of peduncles, 0.3–0.5 cm long, lanceolate. *Flowers* with pedicels 1.5–2.5 cm long, sericeous, with white hairs; sepals 5, ca. 0.5 cm long, elliptic to oblong, margin slightly revolute, apex rounded, adaxial surface glabrescent, abaxial surface white tomentose; glands 4 (–6), 0.5–3 × 0.5–1 mm, prominent, rounded, restricted to sepals, two large, basally fused glands on the dorsal sepals, remaining glands small and free; petals white to light pink, yellow at the base, ca. 1 × 0.8 cm, suborbicular, extremely reflexed, claws ca. 1 mm long. *Stamens* 10, filaments white or light yellow, free or basally fused, 7–13 mm long, glabrous; anthers ca. 0.5 × 0.3 cm, ovate, pubescent, with yellow hairs; pollen sacs dehiscing longitudinally. *Ovary* ca. 2 mm diam.; styles 1, light pink, ca. 13 mm long, curved upwards, deflected either to the left or right side, glabrous; stigma apical. *Mericarps* 3, each flower developing up to three mericarps, detaching from a pyramidal torus; individual mericarps three-winged (laterally placed in the nut), wings pink with greenish base, the posterior wing ca. 3.6 × 1.3 cm, ovoid, apex round or slightly lobed, with white or brown hairs; anterior lateral wings ca. 2.3 × 0.7 cm, lanceolate, arcuate back to the middle; nut ca. 0.2 cm, round or slight ovate, glabrous; areole ca. 0.3–0.6 cm, roughly triangular. *Seeds* angular-globose, ca. 3–5 mm, dark yellow or brown.

**Figure 2.**
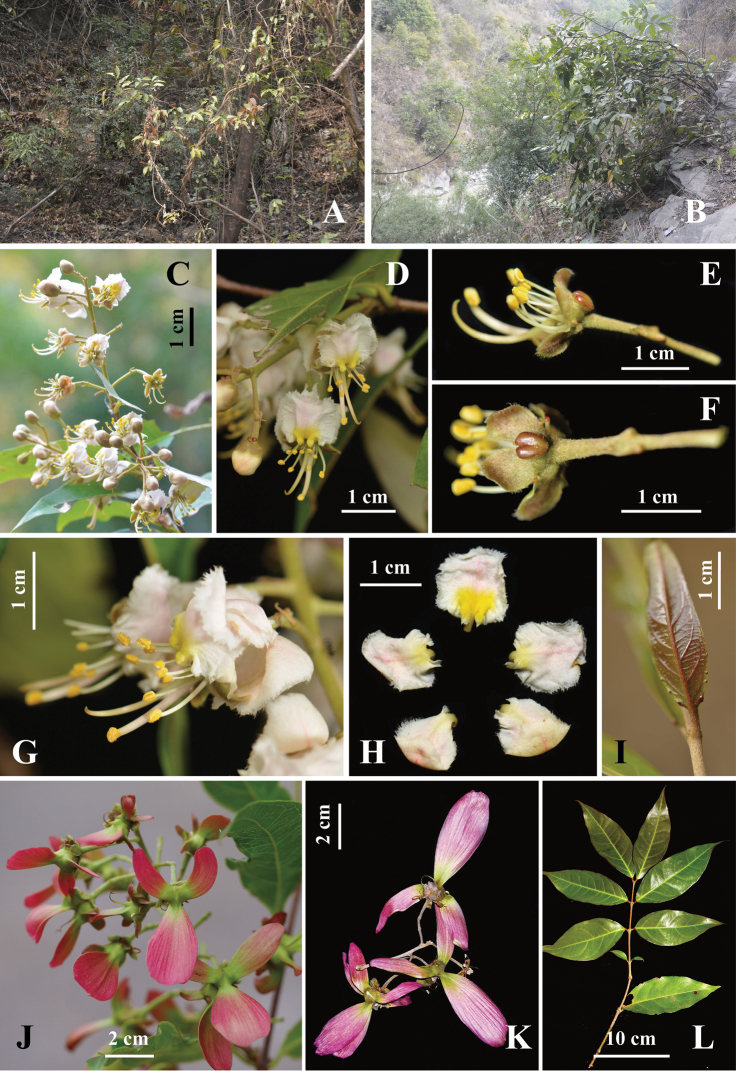
*Hiptage incurvatum* K.Tan & M.X.Ren, sp. nov. **A, B** habit **C** flowering branch **D** flower in frontal view **E** flower with petals removed in sideview **F** flower with petals removed in dorsal view showing two large glands on the dorsal sepals) **G** flowers in sideview **H** detached petals **I** young leaf in adaxial view **J** young samaras **K** mature samaras **L** leaf branch in adaxial view. Photos **A–C** by M. X. Ren, **J, K** by H. L. Zheng and **D–I, L** by K. Tan.

**Figure 3.**
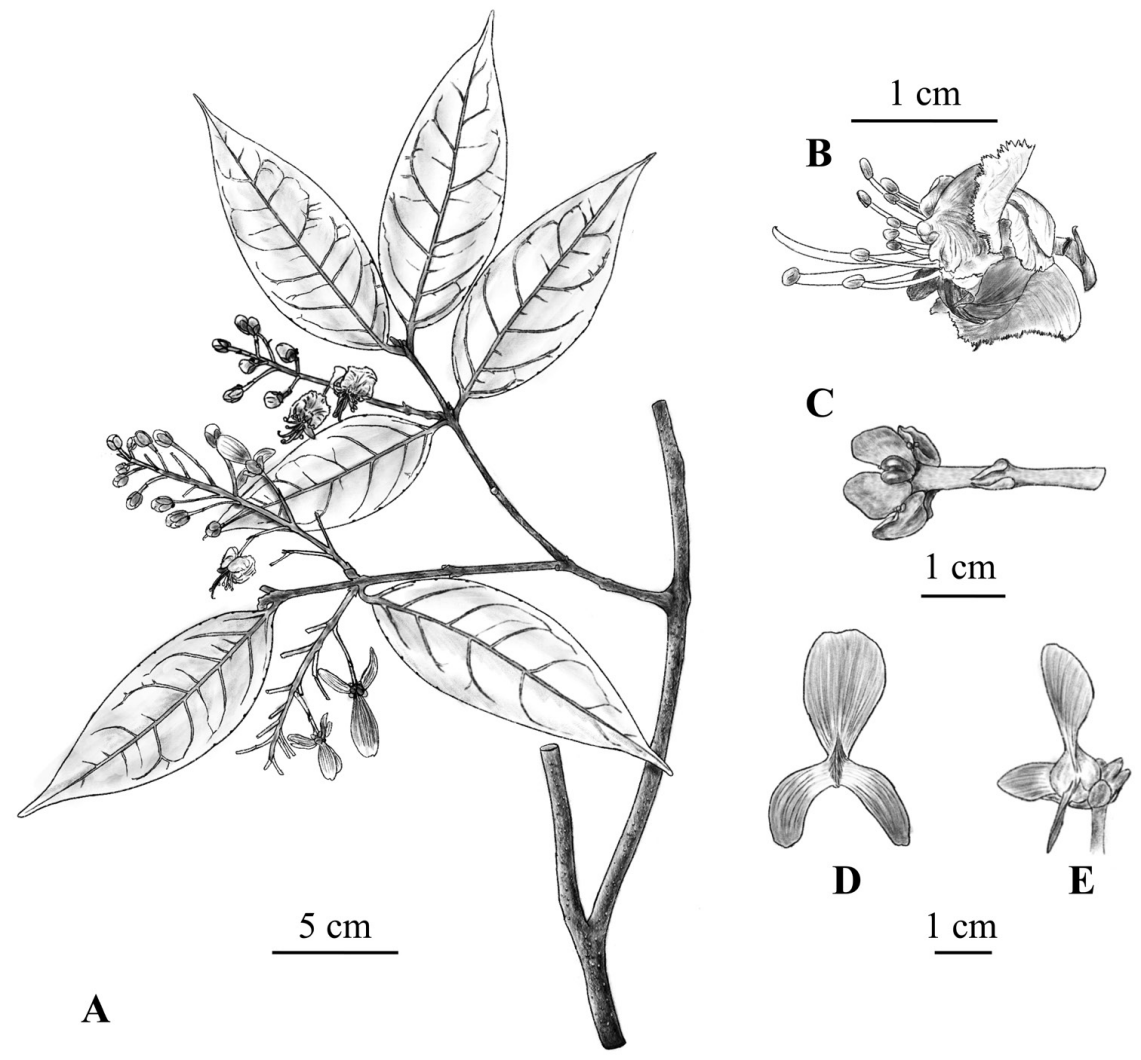
Line drawing of *Hiptage incurvatum* K.Tan & M.X.Ren, sp. nov. **A** flowering branches **B** flower (in sideview) **C** sepals showing two large glands on the dorsal sepals and small glands on the remaining sepals **D** samara in dorsal view, showing the curved lateral wings **E** samara in sideview. Drawings by Ya-Jing Zhang based on K. Tan and M.X. Ren 2019033109 (HUTB).

##### Additional specimens examined (paratypes).

China. Yunnan Province: Pingpo Town, Mt. Cangshan, Dali City. 31 Mar 2019, *K. Tan and M.X. Ren 2019033109* (HUTB), *K. Tan and M.X. Ren 2019033108* (KUN).

##### Phenology.

Flowering from April to May, and fruiting in May.

##### Distribution and habitat.

*Hiptage incurvatum* is only known from two localities near Mt. Cangshan, Pingpo Town, Dali City, North Yunnan, growing on soil slopes or forest margins and river valleys, at 1400–1700 m. In China, a total of 13 species of *Hiptage* now have been recorded, 10 of which, including the new species, are endemic to the country (Chen and Funston 2008; Ren 2015; Yang et al. 2018).

**Figure 4.**
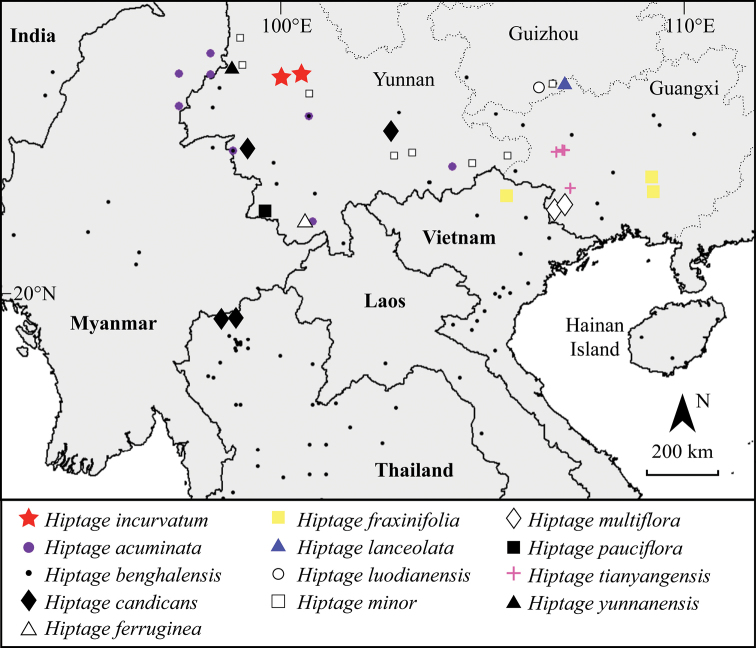
Distribution map of the new species *Hiptage incurvatum* and the other 12 species of the genus known in China and nearby regions.

##### Etymology.

The specific epithet reflects the arcuate and curved anterior lateral wings of the three-winged samara.

##### Vernacular name.

Chinese: 弯翅风筝果 (wān chì fēng zhēng gǔo). The name ‘wān chì’ means arcuate wing, ‘fēng zhēng gǔo’ is the Chinese name of *Hiptage*.

##### Conservation status.

The only two known populations of *Hiptage incurvatum* are in Pingpo Town of Dali City, in a river valley near Mt. Cangshan. These two populations have about 50 individuals in total along the woodland margins or slopes of the valley near a road. Very limited information is known about the new species. Therefore, *H. incurvatum* can be treated as Near Threatened (NT, close to being at high risk of extinction in the near future under the criterion [B1ab(iii) + 2ab (iii)] according to the IUCN Red List criteria (IUCN 2013).

##### Notes.

The new species can be clearly identified from three similar species (*H. benghalensis*, *H. multiflora*, *H. tianyangensis*) from the following traits. Leaf: *H. incurvatum* (ovate, 6–12 × 2.5–4.5 cm) is smaller than all the three species, i.e. *H. tianyangensis* (ovate, 7–12× 3–5.5 cm), *H. multiflora* (oblong, 12–13 × 5–5.5 cm), *H. benghalensis* (elliptic, 9–18 × 3–7cm). Petal color: *H. incurvatum* (white with light pink), *H. tianyangensis* (white), *H. multiflora* (pink), *H. benghalensis* (white with yellow on the vexillum). Calyx glands: *H. incurvatum* (2 large and fused at the lower part, not decurrent to the pedicel; sometimes 2 or 4 smaller glands can be seen on other sepals), *H. tianyangensis* (2, small, clearly isolated, not decurrent to the pedicel), *H. multiflora* (1, large, not decurrent to the pedicel), *H. benghalensis* (1, very large, 1/2 adnate to the pedicel).

### Key to the species of *Hiptage* in China (modified from Chen and Funston 2008)

**Table:** 

1	Calyx eglandular	**2**
–	Calyx glandular	**4**
2	Leaf blades eglandular	***H. lanceolata***
–	Leaf blades with 1 pair of marginal glands near base	**3**
3	Inflorescence covered in yellow-brown appressed hairs; leaf blades ovate, ovate-lanceolate, or elliptic, apex acuminate, base cuneate; petals white, erect	***H. minor***
–	Inflorescence covered in rust-colored hairs; leaf blades elliptic or elliptic-oblong, apex acute to attenuate, base cuneate to obtuse; petals pink to light pink, reflexed	***H. ferruginea***
4	Two or more sepals glandular	**5**
–	Only 1 sepal glandular	**6**
5	Two sepal glands, elliptic; glands slightly adnate to pedicel	***H. luodianensis***
–	4 (-6) sepal glands; rounded; glands restricted to the sepals	***H. incurvatum***
6	Sepal glands rotund or oblong, not decurrent onto the pedicel	**7**
–	Sepal glands oblong, oblong-lanceolate, or ovate-oblong, ± decurrent onto the pedicel	**10**
7	Leaf blade oblong, base cordate, apex acute; posterior lateral wing obovate	**8**
–	Leaf blade elliptic to ovate, base cuneate or rounded, apex acuminate; posterior lateral wing oblong	**9**
8	Basal dotted glands of leaves absent; inﬂorescence with < 10 ﬂowers, pedicels 1.8–2.9 cm, calyx ovate or sub-orbicular to cordate	***H. pauciflora***
–	Basal dotted glands of leaves present, inﬂorescence with >10 ﬂowers, pedicels ca. 1 cm, calyx oblong	***H. multiflora***
9	Thyrses terminal, ca. 11 cm; sepal oblong; leaf base cuneate	***H. fraxinifolia***
–	Thyrses axillary, ca. 3 cm; sepal ovate; leaf base rounded or broadly cuneate	***H. tianyangensis***
10	Leaf blade abaxially yellow-brown or gray-white tomentose; sepal glands oblong-lanceolate, base decurrent onto the pedicel	***H. candicans***
–	Leaf blade glabrous to base of midrib sparsely pubescent abaxially; sepal glands oblong or ovate-oblong, 1/4–1/2 decurrent onto the pedicel	**11**
11	Nut shortly sericeous, wings glabrous; leaf blade oblong, elliptic-oblong, or ovate	***H. benghalensis***
–	Nut and wings pubescent; leaf blade lanceolate, oblong, ovate, or elliptic	**12**
12	Leaf blades lanceolate, oblong, or ovate, 7.5–12 × 3–4.5 cm; posterior lateral wing obovate-oblong, 2.5–3 × ca. 1.2 cm, anterior lateral wing linear-lanceolate, ca. 13 × 5–6 mm	***H. acuminata***
–	Leaf blades elliptic, 12.5–17 × 4–7 cm; posterior lateral wing oblanceolate, ca. 3 × 1 cm, anterior lateral wings linear, ca. 15 × 3 mm.	***H. yunnanensis***

## Discussion

We provide here the first well-sampled phylogenetic study for the Asian endemic *Hiptage*, although this phylogeny is based on a single marker and most clades are not highly supported. *Hiptage* is one of the largest Old-World genera of Malpighiaceae, being adapted to various habitats such as forest edges, river valleys and limestone hills in Asia (Sirirugsa 1991; Ren 2015; Yang et al. 2018). Our phylogenetic tree shows that two species from South Vietnam and Thailand (i.e. *H. stellulifera* and *H. detergens*) were recovered as basal groups, suggesting the genus might have evolved at the southern part of Indo-China Peninsula (Fig. 1).

Based on the phylogeny tree, the most widespread species in the genus, *H. benghalensis*, might have appeared late in the evolution of the genus, although we are not providing divergence time estimates. *H. benghalensis* is well-known for its extremely reflexed petals and single oversized calyx gland secreting nectar, attracting both pollinators and herbivory-defending ants (Ren et al. 2013). Such floral syndromes indicate generalized pollination by pollen-collecting bees (Ren et al. 2013; Qian et al. 2016), which can explain the widespread distribution of *H. benghalensis*. Moreover, both our data and the results of Davis and Anderson (2010) demonstrated the polyphyly in *H. benghalensis* (Fig. 1), suggesting this most widespread species might be treated as two taxa. Further studies are still needed to properly address this question with more extensive sampling.The petal shape and calyx glands are diagnostic traits of the family Malpighiaceae, being used for species identification and taxonomic study (Niedenzu 1924; Anderson et al. 2006; Chen and Funston 2008; Ren 2015). The phylogeny indicates that reflexed petals may be common in both basal and nested clades, and flat petals probably evolved only once (Fig. 1). Normally there are ten oil secreting calyx glands in Neotropical Malpighiaceae, with two glands on each sepal (Anderson et al. 2006). In *Hiptage*, however, most species show a single calyx gland, but secreting nectar instead of oil (Ren et al. 2013). In the basal *H. stellulifera*, five calyx glands were found (i.e., each sepal shows a single gland) (Hô 1992). Therefore, one of the evolutionary trends in *Hiptage* appears to be the numeric reduction of calyx glands (Anderson et al. 2006; Ren et al. 2013; Ren 2015).

The multiple accessions of the proposed new species were recovered as a strongly supported clade (Fig. 1), separated from closely related taxa by weak molecular, but several morphological traits (Fig. 2). Specifically, the new species is distinctive in having two large glands on the dorsal sepals and two small glands on the remaining sepals (Figs 2E–F, 3C). Interestingly, the lower parts of the two large glands are fused (Figs 2F, 3C), indicating a possible explanation for the evolution of the single oversized calyx gland in *H. benghalensis* (Anderson et al. 2006; Ren et al. 2013). The similar evolutionary trend was also found in the Paleotropical genus *Acridocarpus* (Malpighiaceae) (Guesdon et al. 2019), in which the adjacent sepal glands show different degrees of fusion in several species and the single sepal gland in some species shows sagittate-acute shape, shared secretory tissues and vascular bundles, providing strong evidence of the fusion of two glands on adjacent anterior sepals (Vogel 1990; Guesdon et al. 2019).

Molecular data showed that *H. incurvatum* is closely related to *H. tianyangensis*, *H. multiflora* and *H. benghalensis*. These species, however, differ significantly in habitat type, and in calyx gland and mericarp morphology (see Key). The new species grows along a river valley at very high latitudes (>1300 m) in North Yunnan, while *H. multiflora* and *H. tianyangensis* normally grow at the top of limestone mountains in Guangxi and *H. benghalensis* is widespread in Asia in forest margins and riversides (Anderson et al. 2006; Ren 2015).

The most distinctive trait in the new species is the arcuate anterior lateral wings of the three-winged mericarp (Figs 2J–K, 3D). Winged mericarps are an adaption for wind dispersal of fruits (Tan et al. 2018) and the striking diversity of winged mericarps types in Malpighiaceae indicates that this morphology played a role in long-distance dispersals and speciation (Davis et al. 2001, 2002; Qian and Ren 2016; Tan et al. 2018). Pingpo Town is located at the northern edge of the distribution range of the genus *Hiptage*. The surrounding mountains and gorges form a unique isolated habitat, which might be the main reason for the evolution and maintenance of the new species.

## Conclusions

We presented the first well-sampled phylogeny of *Hiptage*, based on the ITS region, suggesting that the southern part of Indo-China Peninsula may be the area of origin of the genus. It also indicates that the erect petals have probably evolved only once in the genus. The number of calyx glands in *Hiptage* seems to have decreased during the genus evolutionary history. And specimens from Mt. Cangshan in North Yunnan were treated as a new species due to forming a highly supported clade in our phylogenetic study and being morphologically distinct from all accepted species in *Hiptage*.

## Supplementary Material

XML Treatment for Hiptage incurvatum
